# Supplementary material for: WordCommentsAnalyzer: A windows software tool for qualitative research
Source: F1000Res. 2018 Sep 4;7:536. Originally published 2018 May 3. [Version 2] doi: 10.12688/f1000research.14819.2 (PMC6137411; doi:10.12688/f1000research.14819.2)
Supplement: Supplementary file 3 [file f1000research-7-17614-s0002.tgz › b2a79496-4ada-47c4-9682-a99ce867836d.zip!/Eight Iranian Health Journals/Archives of Rehabilitation_1.docx]

© Archives of Rehabilitation (RJ). University of Social Welfare and Rehabilitation Sciences, Koodakyar Alley, Daneshjoo Blv., Evin, Tehran, Iran. This work is licensed under [CC BY-NC 4.0](https://creativecommons.org/licenses/by-nc/4.0/).

Volume & Issue: دوره 19 - شماره 1

Title: بررسی و مقایسه رشد جنبه محتوایی مهارت تعریف واژه در دانش‌آموزان 7 تا 12 ساله فارسی‌زبان

http://rehabilitationj.uswr.ac.ir/article-1-1735-fa.html

هدف زبان از سه جزء محتوا، فرم و کاربرد تشکیل شده است که حیطه معناشناختی در زیرمجموعه محتوایی قرار می‌گیرد. در دانش معنایی به اطلاعاتی در زمینه ارتباط هر واژه با دیگر واژه‌های موجود در آن مقوله و مقوله‌های دیگر نیازمندیم. یکی از اجزای اصلی حیطه معناشناختی، دانش واژگان است که بسیاری از ارتباطات واژگانی ازجمله بازنمایی و سازماندهی آن‌ها در این شبکه شکل می‌گیرد. یکی از مفاهیم مهم در حوزه واژگان، سازماندهی ذهنی، ارتباطات و چگونگی شکل‌گیری موقعیت هر یک از واژگان در این شبکه است. یکی از تکالیفی که به سازماندهی واژگان و معنای آن‌ها در شبکه خزانه واژگانی کمک می‌کند تکلیف تعریف واژه است. تعریف واژه مهارتی است که به جنبه‌های مختلفی از رشد زبانی، فرازبانی، سواد و موفقیت‌های تحصیلی مربوط است. از تکلیف تعریف واژه برای بررسی سازماندهی مقوله‌های معنایی در واژگان ذهنی استفاده می‌شود. هدف از این مطالعه بررسی انواع تعریف کردن و نحوه رشد آن در کودکان طبیعی ۷ تا ۱۲ ساله بود که به مدرسه می‌رفتند. روش بررسی پژوهش حاضر از نوع توصیفی تحلیلی است. جامعه آماری شامل دانش‌آموزان طبیعی مقطع دوم تا ششم ابتدایی، از مناطق ۱، ۷ و ۱۷ شهر تهران بودند. نمونه‌ها شامل ۱۵۰ دانش‌آموز بودند که با روش نمونه‌گیری چندمرحله‌ای انتخاب شدند. مهارت تعریف واژه با تکلیف تعریف واژه بررسی شد. در این تکلیف از دانش‌آموزان شرکت‌کننده تقاضا شد تا تعاریف خود را از واژگان بیان کنند. درنهایت تمام پاسخ‌ها و تعاریف، جمع‌آوری و بر اساس محتوا نمره‌دهی شد. پایایی تکلیف تعریف واژه به روش همبستگی نمرات دو ارزشیابی مستقل و روایی آن به صورت محتوایی بررسی شد. تحلیل آماری داده‌های زبانی حاصل از پژوهش با آزمون‌های تی مستقل، تحلیل واریانس و توکی، با استفاده از نرم‌افزار SPSS نسخه ۲۲ انجام شد. یافته‌ها یافته‌های حاصل از این پژوهش حاکی از آن است که با افزایش مقطع تحصیلی و سن دانش‌آموزان، روند رشد تعریف اسامی از طبقه پاسخ‌های عینی و عملکردی، به سمت طبقه پاسخ‌های ترکیبی نوع دوم پیش رفت و میزان استفاده از اصطلاحات سرطبقه، افزایش یافت. در واقع میانگین امتیاز جنبه محتوایی تعریف واژه، افزایش معنی‌داری داشت (۰/۰۰۱>P). همچنین عملکرد دختران در پاسخ‌های محتوایی بهتر از پسران بود و نمرات بالاتری کسب کردند. نتیجه‌گیری نتایج نشان داد با افزایش مقطع تحصیلی و سن دانش‌آموزان، کیفیت پاسخ‌های محتوایی به‌دست‌آمده از بخش محتوایی واژگان افزایش می‌یابد. در واقع، مفاهیم در شبکه معنایی و خزانه واژگان، به صورت سلسله‌مراتبی و شبکه‌ای از ارتباطات پیچیده سازماندهی می شود. همچنین با توجه به طبقه، پاسخ‌ها از مبتدی به پیشرفته و تعاریف از پاسخ‌های عینی و عملکردی به سمت تعاریف ترکیبی نوع دوم و رسمی‌تر پیشرفت می‌کند. در نهایت گفتاردرمان‌ها می‌توانند با توجه به نتایج به‌دست‌آمده از تکلیف تعریف واژه در محیط‌های آموزشی و بالینی استفاده کنند.

Title: تأثیر یک دوره برنامه تمرین‌های نه‌هفته‌ای بر شاخص‌های مرکز فشار در حالت ایستای ساکن با چشمان باز و بسته در مردان سالمند

http://rehabilitationj.uswr.ac.ir/article-1-2119-fa.html

هدف سالمندی با کاهش ظرفیت‌های فیزیولوژیکی و عملکردی ارتباط دارد که می‌تواند باعث ناتوانی، کاهش تعادل و افزایش ریسک سقوط شود. بنابراین از تعادل به عنوان عاملی برای تعیین میزان استقلال سالمندان استفاده می‌شود. از طرفی میانگین سرعت به عنوان پایاترین شاخص مرکز فشار برای ارزیابی تعادل و کاهش ریسک سقوط محسوب می‌شود. با توجه به اهمیت تعادل و قدرت عضلات اندام تحتانی در دوران سالمندی، هدف اصلی پژوهش حاضر بررسی تأثیر یک دوره برنامه تمرین‌های نه‌هفته‌ای بر شاخص‌های مرکز فشار در حالت ایستای ساکن با چشمان باز و بسته در مردان سالمند است. روش بررسی در این پژوهش، ۲۷ مرد سالمند که همگی از نظر عمومی سالم بودند، به صورت داوطلبانه شرکت کردند. شرکت‌کنندگان به صورت تصادفی به دو گروه تمرینی (۱۵ نفر با میانگین سنی ۵/۵۹±۶۱/۰۸ سال، وزن ۱۰/۲۳±۷۷/۰۷ کیلوگرم، قد ۶/۰۴±۱۶۷/۷ سانتی‌متر) و کنترل (۱۲ نفر با میانگین سنی ۶/۴۳±۶۲/۰۸ سال، وزن ۷/۰۶±۷۲/۰۸ کیلوگرم، قد ۹/۰۸±۱۶۷/۰۱ سانتی‌متر) تقسیم شدند. در ابتدای پژوهش، به منظور ارزیابی تعادل، آزمودنی‌ها به حالت ایستاده آرام روی فوت اسکن قرار گرفتند و میانگین سرعت مرکز فشار ابتدا در حالت ایستا با چشمان باز و سپس با چشمان بسته به وسیله دستگاه ثبت شد. داده‌های سینتیکی فوت اسکن (میانگین سرعت مرکز فشار) با نرم‌افزار بالانس آر اس اسکن محاسبه شد. میانگین داده‌ها به صورت فایل اکسل از نرم‌افزار گرفته و ارزیابی شد. سپس گروه تمرینی، برنامه نه‌هفته‌ای تمرین‌های مقاومتی عضلات منتخب اندام تحتانی را (۷ گروه عضلانی اندام تحتانی شامل: دورکننده و نزدیک‌کننده‌های ران، بازکننده‌ها و خم‌کننده‌های ران، بازکننده‌ها و خم‌کننده‌های زانو و بازکننده‌های مچ پا) به مدت یک و نیم ساعت (۱۵ دقیقه گرم‌کردن، ۱ ساعت انجام تمرین اصلی و ۱۵ دقیقه سردکردن) و سه جلسه در هفته انجام دادند. در این مدت از گروه کنترل خواسته شد تا فعالیت‌های روزانه خود را انجام دهند. پس از اتمام برنامه تمرینی مقاومتی از دو گروه پس‌آزمون گرفته شد. داده‌ها با استفاده از تحلیل آماری تی وابسته و مستقل در سطح معنی‌داری (P≤۰/۰۵) تجزیه‌وتحلیل شد. یافته‌ها یافته‌ها نشان داد تمرین‌های مقاومتی عضلات اندام تحتانی به مدت ۹ هفته باعث بهبود معنادار میانگین سرعت مرکز فشار (P=۰/۰۰۵، t=۳/۰۷) و تعادل ایستا با چشمان باز (P=۰/۰۰۴، t=۳/۴۶) در گروه تمرینی نسبت به پیش‌آزمون شده است، اما در حالت تعادل ایستا با چشم‌بسته اثر معناداری یافت نشد (P=۰/۱۵، t=۱/۴۹). همچنین آزمون تی مستقل اختلاف معناداری بین گروه کنترل و تمرینی در میانگین سرعت کلی مرکز فشار (P=۰/۰۴، t=۲/۴۳) و تعادل ایستا با چشمان باز (P=۰/۰۱، t=۲/۳۹) قبل و بعد از تمرین مقاومتی نشان داد. این اختلاف بین دو گروه در حالت تعادل ایستا با چشم‌بسته معنادار نبود (P=۰/۲۲، t=۱/۲۵). نتیجه‌گیری با توجه به اثر مناسب تمرین‌های مقاومتی عضلات اندام تحتانی بر بهبود شاخص مرکز فشار و به دنبال آن بهبود تعادل ایستا و کاهش ریسک سقوط پیشنهاد می‌شود برنامه‌ریزی برای تمرین‌ها و فعالیت‌های جسمانی در جامعه سالمند ضروری شود. با اجرای برنامه تمرین‌های مقاومتی مناسب می‌توان از ضعف تعادلی و ریسک سقوط در این افراد جلوگیری کرد.

Title: بررسی الگوهای عملکرد توجهی در کودکان با اختلال یادگیری ریاضی با استفاده از آزمون «توجه روزمره برای کودکان»

http://rehabilitationj.uswr.ac.ir/article-1-2215-fa.html

هدف اختلال یادگیری ریاضی به عنوان یکی از اختلالات عصبی رشدی با سه نقص حافظه کاری، مشکلات راهبردی و دیداری فضایی قابل تشخیص است. ظرفیت محدود توجه و تعداد بی‌شمار محرکات محیطی، کودکان با اختلالات یادگیری را با مشکلاتی مواجه کرده است. از آنجایی که توجه یکی از اصلی‌ترین عملکردهای شناختی در رشد یادگیری ریاضیات محسوب می‌شود، شناخت و بررسی انواع نقایص توجهی در این گروه از اختلالات اهمیت دارد. بنابراین با در نظر گرفتن مدل سه عاملی مانلی مبنی بر وجود توجه پایدار، توجه انتخابی و کنترل توجه، در مطالعه حاضر به بررسی تفاوت الگوهای توجهی کودکان با اختلال یادگیری ریاضی با استفاده از آزمون توجه روزمره کودکان می‌پردازیم. روش بررسی پژوهش حاضر به عنوان مطالعه‌ای توصیفی‌تحلیلی، روی دانش‌آموزان ۷ تا ۱۱ ساله شهر تهران با اختلال یادگیری ریاضی صورت گرفته است. روان‌پزشک با استناد به تشخیص اولیه اختلال یادگیری ریاضی نمونه‌گیری را انجام داد و نمرات هوش هنجار آزمون وکسلر در پرونده تحصیلی صورت گرفت. با روش نمونه‌گیری در دسترس، ۱۷ نفر از دانش‌آموزان ۷ تا ۱۱ ساله دختر و پسر با مشکلات یادگیری ریاضی از ۵ مرکز دولتی اختلالات یادگیری شهر تهران انتخاب شدند. همچنین ۱۷ کودک عادی از لحاظ سنی و جنسیت همسان‌سازی شدند. ابتدا خانواده‌ها پرسش‌نامه اطلاعات دموگرافیک محقق‌ساخته را تکمیل کردند. سپس محقق ویراست الف از آزمون توجه روزمره برای کودکان را طی مدت زمان استاندارد در مکانی آرام با نور مناسب از دانش‌آموزان گرفت. بر این اساس دو گروه با اختلال یادگیری ریاضی و همتایان سالم در سه حوزه توجه پایدار، انتخابی، کنترل و انتقال توجه ویراست الف آزمون توجه روزمره برای کودکان با یکدیگر مقایسه شدند. یافته‌ها نتایج آزمون شپیراویلک نشان می‌دهد توزیع مقادیر همه متغیرها به غیر از تکلیف دوگانه جست‌وجوی آسمان (توجه پایدار) و سرعت شمارش موجودات (انتقال توجه) در گروه با اختلال یادگیری توزیع نرمالی دارد (P>۰/۰۵) و توجه انتخابی (۰/۰۵>P) در دو گروه تفاوت معناداری را نشان می‌دهند. نتیجه‌گیری نتایج نشان می‌دهد کودکان با اختلال یادگیری ریاضی در هر سه حوزه به میزان قابل ملاحظه‌ای ضعیف‌تر از همتایان سالم عمل کردند. یافته‌ها حاکی از آن است که آزمون توجه روزمره برای کودکان قابلیت احتمالی تشخیص نقایص توجهی اختلال یادگیری ریاضی را در مقایسه با همتایان سالم دارد. بنابراین می‌تواند ابزار مناسبی برای ارزیابی در این گروه محسوب شود.
